# Comparison and combination of blood-based inflammatory markers with faecal occult blood tests for non-invasive colorectal cancer screening

**DOI:** 10.1038/bjc.2012.104

**Published:** 2012-03-27

**Authors:** S Tao, U Haug, K Kuhn, H Brenner

**Affiliations:** 1 Division of Clinical Epidemiology and Aging Research (C070), German Cancer Research Centre (DKFZ), Im Neuenheimer Feld 581, 69120 Heidelberg, Germany; 2 Division of Preventive Oncology, National Centre for Tumor Diseases, German Cancer Research Centre (DKFZ), Im Neuenheimer Feld 460, 69120 Heidelberg, Germany

**Keywords:** inflammatory markers, faecal occult blood tests, colorectal cancer screening

## Abstract

**Background:**

Faecal occult blood tests (FOBTs) are widely used for colorectal cancer (CRC) screening. Blood-based inflammatory markers have been suggested as alternative or supplementary non-invasive CRC screening tests.

**Methods:**

Among 179 CRC patients, 193 people with advanced adenoma and 225 people free of neoplasm, C-reactive protein (CRP), serum CD26 (sCD26), complement C3a anaphylatoxin and tissue inhibitor of metalloproteinases 1 (TIMP-1) levels in blood were measured by ELISA tests, and an immunochemical FOBT (iFOBT) and a guaiac-based FOBT were performed. Receiver operating characteristic curves were constructed and the areas under the curves (AUCs) were compared.

**Results:**

The blood levels of CRP, sCD26 and TIMP-1 showed statistically significant differences between CRC patients and neoplasm-free participants, and levels of TIMP-1 were furthermore significantly elevated in advanced adenoma patients. For the four inflammatory markers, AUCs ranged from 0.52 to 0.62 for CRC detection and from 0.50 to 0.58 for advanced adenomas detection, compared with AUCs of 0.90 and 0.68 for the iFOBT. At 97% specificity, blood markers showed much lower sensitivities than FOBTs. Combining inflammatory markers with the iFOBT increased the AUC for advanced adenomas.

**Conclusion:**

These blood-based markers do not seem to be an alternative to FOBT-based CRC screening. The potential use of these and other blood-based tests in combination with iFOBT might deserve further attention.

With more than 1.2 million new cases and more than 600 000 deaths annually, colorectal cancer (CRC) is the third most common cancer and the fourth most common cancer cause of death globally (Ferlay *et al*, 2008). Because of its typically slow development, perspectives for prevention and early detection by screening are much better than for most other forms of cancer. Several randomised controlled trials have demonstrated that CRC incidence and mortality can be reduced by screening with faecal occult blood tests (FOBTs) (Hewitson *et al*, 2008). However, FOBTs have low sensitivity for detecting colorectal neoplasms, especially precursors of CRC (colorectal adenomas). Although immunochemical FOBTs (iFOBTs) showed more promising test characteristics than the traditional guaiac-based FOBTs (gFOBTs) (Guittet *et al*, 2007; van Rossum *et al*, 2008; Hol *et al*, 2009; Hundt *et al*, 2009; Park *et al*, 2010), further improvement of non-invasive tests for early detection of colorectal neoplasms is highly desirable.

Several recent studies have suggested that blood-based inflammatory markers, such as C-reactive protein (CRP), serum CD26 (sCD26) and complement C3a anaphylatoxin (C3a), may have potential to complement CRC screening methods (Habermann *et al*, 2006; De Chiara *et al*, 2010; Kwon *et al*, 2010; Nielsen *et al*, 2011). However, no previous study has compared the performance of inflammatory markers with FOBTs directly and assessed the potential benefit of combining these tests for the detection of CRCs and advanced adenomas so far.

We aimed to determine the diagnostic performance of selected blood-based inflammatory markers in comparison and combination with FOBTs for CRC screening.

## Methods

### Study population

The study population consisted of participants recruited in the context of the BliTz study (Begleitende Evaluierung innovativer Testverfahren zur Darmkrebsfrüherkennung), and a satellite sub-study (DACHS+) to the DACHS study (Darmkrebs: Chancen der Verhütungdurch Screening). The latter is an ongoing case-control study focusing on the role of colonoscopy in CRC prevention (Brenner *et al*, 2006; Brenner *et al*, 2007; Brenner *et al*, 2011).

Detailed information on the BliTz study has been reported elsewhere (Hundt *et al*, 2009; Brenner *et al*, 2010a, 2010b, 2010c; Haug *et al*, 2010). Briefly, it is an ongoing prospective screening study conducted in cooperation with 20 gastroenterology practices in south-western Germany and initiated in 2005. Participants are recruited at a preparatory visit of a screening colonoscopy, which is offered as a primary screening examination to the resident population aged 55 years or older in Germany since October 2002. This analysis includes participants recruited between 2005 and 2008. We excluded participants with inflammatory bowel disease, participants who had a colonoscopy in the past 5 years and participants with visible blood in stool (*n*=290). Further exclusion criteria were inadequate bowel preparation (*n*=98), incomplete colonoscopy (*n*=28), and lack of histological classification of polyps detected at colonoscopy (*n*=71). As we focused on assessing the sensitivity for the detection of CRC and advanced colorectal adenomas, as well as specificity among subjects free of neoplasms, we further excluded participants with non-advanced adenomas (*n*=401) or with hyperplastic polyps only (*n*=198). Because of limited resources, the tests were not performed in all participants from the large group free of findings at colonoscopy (*n*=1198). Rather, we randomly selected 225 participants and included them in this analysis. This sample size still ensured reasonably high levels of precision of estimates of specificity.

Participants from the DACHS+ study were CRC patients referred by general practitioners or gastroenterologists for surgery to one of four participating hospitals between 2006 and 2008. Patients who received neoadjuvant therapy before sample collection (*n*=66) were excluded from this study. Both studies were approved by the Ethics committee of the University of Heidelberg.

### Sample collection and laboratory measurements

In the BliTz study, blood and stool samples were collected before bowel preparation for colonoscopy. In the DACHS+ study, the stool samples were collected before hospitalisation for large bowel surgery, in 75% of cases more than 1 week after the last colonoscopy. The blood samples in the DACHS+ study were collected after hospitalisation but before surgery. Details on standard operational procedures for stool sample collection were described previously (Hundt *et al*, 2009; Haug *et al*, 2010). Briefly, all participants received a small stool container (60 ml) to collect a stool sample from one bowel movement and kept it in a provided plastic bag in the freezer or, if not possible, in the refrigerator at home until return to the practice or hospital. Blood and stool samples were transported to the central laboratory in a cool chain, where blood samples were centrifuged at 2123 **g** for 10 min and stored at −80 °C until analyses. Serum concentrations of CRP, sCD26 and C3a and plasma concentration of tissue inhibitor of metalloproteinase 1(TIMP-1) were measured with commercial kits CRP, sCD26, TIMP-1: Bender Medsystems (Vienna, Austria); C3a: BD Biosciences (San Diego, CA, USA) according to the manufacturers’ instructions. An automated ELISA-based quantitative iFOBT (RIDASCREEN Haemoglobin, R-Biopharm AG, Bensheim, Germany) was performed to measure haemoglobin levels in stool. The lower detection limit of this test is 0.42 *μ*g g^−1^ stool, and the cutoff point for test positivity given by the manufacture is of 2 *μ*g g^−1^ stool. A qualitative gFOBT (HemOccult, Beckman Coulter, Krefeld, Germany) was rated (positive or negative) by physician assistants. Both stool tests were based on a single bowel movement. All blood and stool tests were carried out blinded with respect to diagnosis.

After colonoscopy or surgery, colonoscopy and histology reports were collected from the practices or participating hospitals. Relevant information was extracted in a standardised manner by two independent trained research assistants who were blinded to blood and stool test results. Advanced adenomas were defined as adenomas with at least one of the following features: ⩾1 cm in size, tubulovillous or villous components, high-grade dysplasia. Cancer stages were classified according to the UICC classification. Because of the low number of stage IV cancers, stage III and stage IV cancers were combined in a common category ‘advanced stage’ cancers.

### Statistical methods

The three groups of participants (those with no neoplasm, those with advanced adenoma and those with CRC) were described with respect to socio-demographic characteristics. Medians and interquartile ranges of marker concentrations were calculated for each of the groups. Potential variation of the inflammatory markers by sex and age were assessed by the Wilcoxon rank-sum test and the Spearman rank correlation coefficient, respectively, within the group of neoplasm-free participants. Primary study endpoints were differences in blood levels of the four inflammatory markers between participants with no neoplasms and those with advanced adenoma and CRC (overall and by stage). Medians and interquartile ranges of the four inflammatory markers were calculated for the different groups, and differences were tested for statistical significance by the Wilcoxon rank-sum test.

In addition, sensitivities were calculated at cutoff points yielding the level of specificity observed for gFOBT, the current standard test applied in Germany (defined as the proportion of negative results among participants free of neoplasms from the same study population), to facilitate the comparison of the test sensitivities. Further analyses were conducted on differences of test performance in CRC patients according to cancer stage, location and recruitment settings. Receiver operating characteristic (ROC) curves of the four blood-based markers and iFOBT results were constructed and the areas under the curves (AUCs) with their corresponding 95% CIs were calculated using the method described by DeLong *et al* (1988).

For the iFOBT and blood markers whose levels showed statistically significant differences in CRC cases *vs* participants free of neoplasms, additional analyses by logistic regression were carried out. No other covariates were included in the regression models. Discriminative probabilities of presence of advanced adenomas *vs* absence of any neoplasms and of presence of CRC *vs* absence of any neoplasms were determined in separate logistic regression models by the C-statistic, which is equivalent to the AUC of the ROC curve. We further assessed the AUC for models including combinations of iFOBT and the blood markers, as well as *P*-values of likelihood ratio tests for adding the blood markers to iFOBT-based models.

Statistical analyses were performed with the SAS package version 9.2 (SAS Institute, Cary, NC, USA). Receiver operating characteristic curves were derived by using MedCalc for Windows, version 9.6.4.0 (MedCal Software, Mariakerke, Belgium).

## Results

Overall, 597 participants were included in our analyses; among these were 179 patients with CRC, 193 with advanced adenoma and 225 participants free of colorectal neoplasm. Whereas blood samples were available for all participants, availability of stool samples is restricted to 67 CRC patients, 183 patients with advanced adenoma and 217 participants free of neoplasms. Stool samples were often missing for CRC patients recruited in the clinical setting because they could often not be obtained before initiation of therapy. There was no significant difference in CRC patients with and without stool samples with respect to age, stage and cancer location (data not shown).

The distribution of socio-demographic characteristics among the three diagnostic subgroups and the stage distribution of CRCs are shown in Table 1. More than half of patients with advanced adenoma or CRC were men, compared with 44.4% of those free of colorectal neoplasms. Mean age was highest among patients with CRC (68.1 years), intermediate among patients with advanced adenomas (65.0 years) and lowest among participants free of colorectal neoplasms (61.9 years). None of the blood markers showed any relevant or statistically significant relationship with sex or age within the group of participants free of neoplasms (all *P*-values >0.05, data not shown).

**Figure 1:**
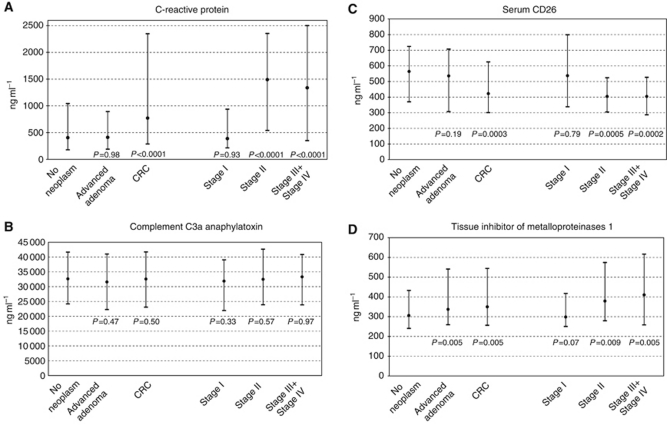
Comparison of blood levels (median and interquartile range) of inflammatory markers in different groups (*P*-values refer to comparison with participants free of neoplasm). (**A**) C-reactive protein (CRP). (**B**) Complement C3a anaphylatoxin (C3a). (**C**) Serum CD26 (sCD26). (**D**) Tissue inhibitor of metalloproteinases 1 (TIMP-1). Abbreviation: CRC=colorectal cancer.

Medians and interquartile ranges of the blood marker levels stratified by diagnostic subgroups (with further stratification of CRC patients by stage of disease) are shown and compared in Figure 1. The TIMP-1 levels were found to be elevated in both advanced adenoma and CRC patients compared with neoplasm-free participants (*P*=0.005 for each comparison). There was also a tentative positive association between increased blood levels of TIMP-1 and more advanced CRC stages. Concentrations of CRP were significantly higher and concentrations of sCD26 were significantly lower in CRC patients compared with neoplasm-free participants (*P*<0.0001 and *P*=0.0003, respectively). However, CRP elevations and sCD26 reductions were essentially restricted to stages II, III and IV CRC, and no significant differences were seen between patients with advanced adenomas and participants free of colorectal neoplasms. For C3a, no significant differences were observed between any of the diagnostic subgroups.

**Figure 2:**
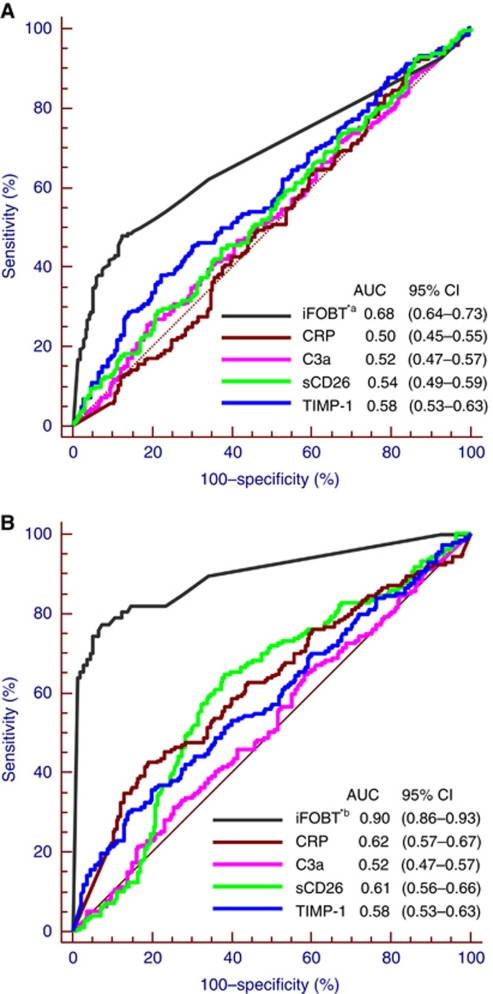
Receiver operating characteristics curves of different inflammatory markers and the iFOBT (**A**) for the detection of advanced adenoma. *^a^ Results pertain to participants with available stool samples (217 without neoplasm and 183 with advanced adenoma); (**B**) for the detection of colorectal cancer. *^b^ Results pertain to participants with available stool samples (217 without neoplasm and 67 with colorectal cancer). Abbreviations: AUC=area under the curve; CI=confidence interval; iFOBT=immunochemical faecal occult blood test; CRP=C-reactive protein; C3a=complement C3a anaphylatoxin; sCD26=serum CD26; TIMP-1=tissue inhibitor of metalloproteinases 1.

Receiver operating characteristic curves for detecting advanced adenoma and CRC of the four blood-based markers and the iFOBT are shown in Figures 2A and B, respectively. Among inflammatory markers, AUCs ranged from 0.50 to 0.58 for detection of advanced adenomas and from 0.52 to 0.62 for detection of CRC. Tissue inhibitor of metalloproteinases-1 showed the largest AUC for detection of advanced adenoma and CRP showed the largest AUC for detection of CRC among the four tested blood markers. The AUCs of the iFOBT are estimated to be 0.68 and 0.90 for detection of advanced adenoma and CRC, respectively.

Based on 217 participants free of neoplasms, 183 advanced adenoma patients and 67 CRC patients, for whom both blood and stool tests could be performed, sensitivities at cutoff points yielding 97.7% specificity, corresponding to the level of specificity observed for the gFOBT, are shown in Table 2. The iFOBT showed the by far highest sensitivity (19.7% and 65.7% for detecting advanced adenoma and CRC, respectively, at a cutoff point yielding 97.7% specificity). These sensitivities were much higher than those of the gFOBT (7.7% and 40.3%, respectively). Sensitivities of the blood-based inflammatory markers were lowest and even far below those of the gFOBT in most cases. Given that the upper limit of CRP measurements was 2500 ng ml^−1^, the specificity was not able to reach 97.7% for comparison at any cutoff points. Even the combination of the results of the blood-based markers in an algorithm determined by multiple logistic regression yielded sensitivities far below those observed for the FOBTs (5.5% and 19.4% for advanced adenoma and CRC, respectively at a cut point yielding 97% specificity; data not shown in the table).

Test sensitivities for detection of CRC stratified by cancer stage, location and recruitment setting were further assessed based on participants with available stool samples at cutoffs yielding 97% specificity (Table 3). The sensitivity of gFOBT and iFOBT were found to be significantly related to CRC stage (*P*=0.003 and *P*=0.001, respectively), and sensitivity of iFOBT was significantly higher for rectal cancer than for colon cancer (*P*=0.03). No significant differences were seen by setting of recruitment (clinical setting *vs* screening colonoscopy).

Different combinations of blood-based markers (CRP, sCD26 and TIMP-1) with iFOBT were entered in logistic regression models predicting presence of advanced adenoma or CRC based on participants with available stool samples, and the corresponding AUC as well as *P*-values for adding the blood-based markers to the iFOBT model were calculated. For advanced adenoma, the model fit was significantly improved only by adding TIMP-1 or all three blood tests (*P*=0.002 and *P*=0.0007, respectively) according to likelihood ratio tests. The AUC only increased from 0.683 for a model including iFOBT alone to 0.710 and 0.729 by adding TIMP-1 or all three blood tests, respectively. At a cutoff point yielding 97.7% specificity, sensitivities for detection of advanced adenoma were 21.3% and 21.9% by combining TIMP-1 or all three blood markers with iFOBT, respectively, compared with 19.7% for a model based on iFOBT alone. Adding the blood markers did not lead to any meaningful improvement of the detection of CRC.

## Discussion

In this article, we determined the blood levels of four inflammatory markers and assessed their potential in discriminating patients with CRC and advanced adenomas from participants free of colorectal neoplasm in comparison and combination with FOBTs. C-reactive protein, sCD26 and TIMP-1 levels were found to be different in CRC patients compared with participants free of neoplasms. Concentrations of TIMP-1 were also significantly elevated in advanced adenoma patients. However, the diagnostic performance of these four blood markers was worse than performance of FOBTs, both regarding the detection of advanced adenomas (for which performance was hardly any better than chance) and CRC. Combinations of iFOBT and blood markers suggested some at best modest improvements in detection of advanced adenomas.

Our results regarding the differences in blood levels of CRP, sCD26 and TIMP-1 among CRC patients *vs* neoplasm-free participants are in line with those of several previous studies (Holten-Andersen *et al*, 1999; Cordero *et al*, 2000; Holten-Andersen *et al*, 2002; Holten-Andersen *et al*, 2004; Nikiteas *et al*, 2005; De Chiara *et al*, 2010; Kwon *et al*, 2010; Shimwell *et al*, 2010). Elevated blood levels of C3a in CRC patients have been reported in one study (Habermann *et al*, 2006), but could not be confirmed in our analysis. For detection of advanced adenoma, TIMP-1 (Holten-Andersen *et al*, 2004; Nielsen *et al*, 2011) and sCD26 (De Chiara *et al*, 2010) levels have been analysed previously. In agreement with our results, Nielsen *et al* (2011) recently reported significantly elevated TIMP-1 concentration in 856 adenoma patients compared with in 1176 participants without neoplastic findings (verified by colonoscopy or sigmoidoscopy). However, no difference could be detected according to size of adenomas (⩾1 cm *vs* 1 cm); and no such association had been found in a previous, much smaller study (Holten-Andersen *et al*, 2004). A sensitivity of 41.7% at a cutoff yielding 79.4% specificity of sCD26 was reported by De Chiara *et al* (2010).

Diagnostic characteristics of sCD26 and TIMP-1, for the detection of CRC were reported by three studies for each of the two markers. The reported performance characteristics of sCD26 varied greatly and were mostly better than we observed in this analysis. De Chiara *et al* (2010) recently reported 81.8% sensitivity at a cutoff point yielding 72.3% specificity for CRC detection by ELISA-based sCD26 testing, based on 33 CRC patients and 68 symptomatic participants without colorectal pathology. They also reported a higher AUC than we found (0.81 *vs* 0.61). Even better performance of sCD26, with 90% sensitivity at a cutoff point yielding 90% specificity for CRC detection had previously been reported in a study on 110 CRC patients and 52 healthy blood donors from Spain (Cordero *et al*, 2000). Regarding the performance of TIMP-1, Wild *et al* (2010) conducted a study with a similar design as ours, in which most of the neoplasm-free participants were recruited in a screening setting and CRC patients were mostly from clinical settings. They reported a sensitivity of 26.8% at a cutoff point yielding 95% specificity, which is similar to our results (13.4% sensitivity at a specificity of 98%), even though, as shown in Figure 2B, we observed a slightly inferior AUC level (0.58 *vs* 0.66) for CRC detection. In a Danish study, the AUC for TIMP-1 testing was reported to be 0.70 regarding CRC detection in a group of high-risk individuals (Nielsen *et al*, 2011).

To our knowledge, our study is the first to evaluate blood-based inflammatory markers in comparison and combination with FOBTs directly. Test performance of these markers was much worse than performances of FOBTs, especially compared with the iFOBT, a benchmark for non-invasive CRC screening so far. At cutoff points yielding the level of specificity of gFOBT (97.7%), sensitivities of the four blood markers were all less than 20% for detection of CRC compared with 40% of gFOBT and 66% for iFOBT. Sensitivities for advanced adenomas were likewise much lower for the blood based tests than for iFOBT. Comprehensive ROC analyses confirmed these patterns for a wide range of cutoff points. Our results suggest potential modest benefits of combining the blood-based inflammatory markers with iFOBT for detection of advanced adenomas, but not for detection of CRC. However, the potential modest benefits for detecting advanced adenomas seem to be of very limited clinical relevance and would not justify the additional complexity and costs of combining blood and stool tests, which may also go along with reduced compliance compared with testing with iFOBT alone. Comparable improvements in the AUC could be achieved by adding other more easily measured variables, such as age and sex (data not shown). Nevertheless, the possible merits of test combinations in novel multiple test approaches including additional serological markers, might be a promising area for further research.

Some specific strengths and limitations of our study deserve careful consideration. All participants included in this analysis had undergone colonoscopy, which minimises potential misclassification by inclusion of carriers of undetected adenomas or CRC in the control group. In addition, neoplasm-free participants and patients with advanced adenomas were recruited from a true screening setting, that is, in a population that represents the target population for application of screening tests. Furthermore, multiple blood tests and stool-based tests were applied in the same individuals, which allowed direct comparison and combination of different tests. Because of the small numbers of CRC patients in the study population recruited in the screening setting, we had to rely on a study recruiting CRC patients after diagnosis (but before hospitalisation and neoadjuvant or other therapy) to assess sensitivity of the test for detecting CRC. The proportion of early stage (stage I or II) cancer in this population was somewhat lower than the proportion expected in a true screening setting, which may have led to slight overestimation of overall levels of sensitivity, a limitation that is shared with most other pertinent studies assessing diagnostic performance of potential CRC screening markers (Hundt *et al*, 2007; Tao *et al*, 2011). Our study is further limited by the exclusion of people with non-advanced adenoma and hyperplastic polyps in the group of neoplasm-free participants, which is expected to lower test specificity at given cutoff points. However, potential bias from this source appears to be small, given that very low positivity rates of all blood-based tests even for advanced adenomas (see Table 2). Model calculations assuming analogous positivity rates among carriers of non-advanced adenoma and hyperplastic polyps suggest expected changes in specificity ranging from 0.5 to 1.6% compared with those shown in Table 2, if carriers of non-advanced adenomas and hyperplastic polyps had been included.

Taken together, our results showed that blood levels of sCD26, CRP and TIMP-1 were different in CRC patients compared with participants free of neoplasms, and TIMP-1 levels were also elevated in advanced adenoma patients. However, the diagnostic performance of the four blood markers was clearly worse than performance of iFOBT for detecting advanced adenoma and CRC. Although the combination of these blood markers with the iFOBT slightly improved detection of advanced adenomas, the blood markers of inflammation assessed in our analysis do not seem to be an alternative for non-invasive CRC screening. Nevertheless, the potential use of multiple blood-based markers (including and beyond those assessed in this study) in combination with iFOBT using novel multiplex laboratory technologies might deserve further attention.

## Figures and Tables

**Table 1:** Characteristics of the study population according to diagnostic subgroup

			**Colorectal cancer** ***N*****(%)**		
	**No colorectal neoplasm** ***N*****(%)**	**Advanced adenoma** ***N*****(%)**	**Screening setting**	**Clinical setting**	**Total**
*Sex*					
Female	125 (55.6)	69 (35.8)	5 (35.7)	76 (46.1)	81 (45.3)
Male	100 (44.4)	124 (64.3)	9 (64.3)	89 (53.9)	98 (54.8)

*Age*					
<60 years	89 (39.6)	52 (26.9)	2 (14.3)	36 (21.8)	38 (21.2)
60–69 years	104 (46.2)	93 (48.2)	8 (57.1)	52 (31.5)	60 (33.5)
>70 years	32 (14.2)	48 (24.9)	4 (28.6)	77 (46.7)	81 (45.3)

*Stage*					
I			7 (50.0)	49 (29.7)	56 (31.3)
II			1 (7.1)	48 (29.1)	49 (27.4)
Advanced (III+IV)			5 (35.7)	67 (40.6)	72 (40.2)
Missing			1 (7.1)	1 (0.6)	2 (1.1)

*Location*					
Colon			7 (50.0)	105 (63.6)	112 (62.7)
Rectum			6 (42.9)	59 (35.8)	65 (36.3)
Missing			1 (7.1)	1 (0.6)	2 (1.1)

Total	225	193	14	165	179

**Table 2:** Diagnostic performance of gFOBT, iFOBT and blood-based inflammatory markers: sensitivities and specificities for detection of advanced adenoma and colorectal cancer (CRC)

		**Sensitivity % (95% CI)**		
**Test**	**Cut point (ng ml** **^−1^)**	**Advanced adenoma (** ***n*****=183)**	**CRC (** ***n*****=67)**	**Specificity % (95% CI)**
gFOBT	—	7.7 (4.6–12.4)	40.3 (29.4–52.3)	97.7 (94.7–99.0)
CRP	2499.9	4.9 (2.6–9.1)	19.4 (11.7–30.4)	90.3 (85.7–93.6)
sCD26	133.5	4.4 (2.2–8.4)	0 (0–5.4)	97.2 (94.1–98.7)
TIMP-1	928.2	7.1 (4.2–11.8)	13.4 (7.2–26.6)	97.7 (94.7–99.0)
iFOBT	24.0^a^	19.7 (14.6–26.0)	65.7 (53.7–75.9)	97.7 (94.7–99.0)

Abbreviations: CI=confidence interval; CRP=C-reactive protein; gFOBT=guaiac-based faecal occult blood test; iFOBT=immunochemical faecal occult blood test; sCD26=serum CD26; TIMP-1=tissue inhibitor of metalloproteinases 1.

a *μ*g per gram stool.

**Table 3:** Sensitivities of FOBTs and blood-based inflammatory markers at given levels specificity for detection of colorectal cancer stratified by stage, location and recruitment setting

		**Sensitivity at cutoff yielded 97.7% specificity % (95% CI)**				
	**Number**	**gFOBT**	**CRP^a^**	**sCD26**	**TIMP-1**	**iFOBT**
*Stage*						
I	21	14.3 (5.0–34.6)^b^	9.5 (2.7–28.9)	0 (0–15.5)	4.8 (0.8–22.7)	33.3 (17.2–54.6)^b^
II	16	37.5 (18.5–61.4)^b^	12.5 (3.5–36.0)	0 (0–19.4)	25 (10.2–49.5)	75.0 (50.5–89.8)^b^
Advanced stage	29	62.1 (44.0–77.3)^b^	31.0 (17.3–49.2)	0 (0–11.7)	13.8 (5.5–30.6)	82.8 (64.5–92.4)^b^

*Location*						
Colon	40	45.0 (30.7–60.2)	22.5 (12.3–37.5)	0 (0–8.8)	17.5 (8.7–32.0)	55.0 (39.8–69.3)^b^
Rectum	26	30.8 (16.5–50.0)	11.5 (4.0–29.0)	0 (0–12.9)	7.7 (2.1–24.1)	80.8 (62.1–91.5)^b^

*Recruitment setting*						
Screening setting	13	23.1 (8.2–50.3)	15.4 (4.3–42.2)	0 (0–22.8)	0 (0–22.8)	69.2 (42.4–87.3)
Clinical setting	54	44.4 (32.6–62.0)	20.4 (11.8–32.9)	0 (0–6.6)	16.7 (9.0–28.7)	64.8 (51.5–76.2)

Abbreviations: CI=confidence interval; CRP=C-reactive protein; gFOBT=guaiac-based faecal occult blood test; iFOBT=immunochemical faecal occult blood test; sCD26=serum CD26; TIMP-1=tissue inhibitor of metalloproteinases 1.

a Sensitivity at cutoff yielding 90% specificity.

b *P*-value <0.05 by Chi-square or Fisher exact test.
